# Efficient production of chimeric mice from embryonic stem cells injected into 4- to 8-cell and blastocyst embryos

**DOI:** 10.1186/2049-1891-4-12

**Published:** 2013-03-21

**Authors:** Minhua Hu, Hengxi Wei, Jingfeng Zhang, Yinshan Bai, Fenglei Gao, Li Li, Shouquan Zhang

**Affiliations:** 1 Agricultural Animal Genomics and Molecular Breeding Key Lab of Guangdong, Province, College of Animal Science, South China Agricultural University, Guangzhou, 510642, China

**Keywords:** Chimeric mice, Embryonic stem cell, Microinjection

## Abstract

**Background:**

Production of chimeric mice is a useful tool for the elucidation of gene function. After successful isolation of embryonic stem (ES) cell lines, there are many methods for producing chimeras, including co-culture with the embryos, microinjection of the ES cells into pre-implantation embryos, and use of tetraploid embryos to generate the full ES-derived transgenic mice. Here, we aimed to generate the transgenic ES cell line, compare the production efficiency of chimeric mice and its proportion to yield the male chimeric mice by microinjected ES cells into 4- to 8-cell and blastocysts embryos with the application of Piezo-Micromanipulator (PMM), and trace the fate of the injected ES cells.

**Results:**

We successfully generated a transgenic ES cell line and proved that this cell line still maintained pluripotency. Although we achieved a satisfactory chimeric mice rate, there was no significant difference in the production of chimeric mice using the two different methods, but the proportion of the male chimeric mice in the 4- to 8-cell group was higher than in the blastocyst group. We also found that there was no tendency for ES cells to aggregate into the inner cell mass using *in vitro* culture of the chimeric embryos, indicating that they aggregated randomly.

**Conclusions:**

These results showed that the PMM method is a convenient way to generate chimeric mice and microinjection of ES cells into 4- to 8-cell embryos can increase the chance of yielding male chimeras compared to the blastocyst injection. These results provide useful data in transgenic research mediated by ES cells.

## Background

Embryonic stem (ES) cells derived from the inner cell mass (ICM) of blastocysts can maintain their self-renewal during *in vitro* culture and have the amazing ability of developing into all three germ layers, including germ cells [1]. The ES cells can be genetically manipulated *in vitro* by introducing targeted mutations and other genetic alterations into the mice genome, providing a powerful tool for understanding gene function *in vivo*[1-7] and contributing significantly to biomedical research [8,9]. Due to the powerful function of ES cells, chimeras have become important tools for the study of cell lineage differentiation and embryogenesis. In 1961, the first chimeric mice were generated by aggregation of two 8-cell stage embryos [10]. Since then, different methods of producing chimeric mice and more efficient equipment have been created. Until now, the production of chimeras by microinjected genetically modified ES cells into blastocysts or zygotes [11,12] or 8-cell stage embryos [13] was successful. In addition, chimeric mice can also be produced by aggregation of ES cells with cleavage embryos [14-16]. The Piezo-Micromanipulator (PMM) method, which originally was successfully used for intracytoplasmic sperm injection (ICSI) and somatic cell cloning [17,18], was also used in chimeric mice production [19]. Laser-assistance of injecting ES cells into 8-cell stage embryos could more efficiently produce F0 ES mice with full germ-line transmission compared to the tetraploid complementation method [20]. Recently, Huang report that injection of ES cells into 4- or 8-cell embryos with the application of PMM could directly produce the F0 ES cell offspring [21].

In the present study, we generated an EGFP-ES cell line and tested the efficiency of producing chimeric mice by injecting the ES cells into 4- to 8-cell and blastocyst embryos with the application of PMM. The proportion of male chimeric mice and *in vitro* development of the chimeric embryos was also analyzed.

## Methods

### Embryos and recipient mice

All animal procedures were performed according to guidelines developed by the China Council on Animal Care and protocols were approved by the Animal Care and Use Committee of Guangdong Province, China. The approval ID or permit numbers are SCXK (Guangdong) 2004–0011 and SYXK (Guangdong) 2007–0081.

CD-1 females were superovulated by intraperitoneal injection of 5 IU pregnant mare serum gonadotrophin followed by 5 IU human chorionic gonadotrophin (HCG) 46 to 48 h later. After the HCG injection, these female mice were mated with male mice of the same strain. Females were screened for vaginal plugs the following morning (0.5 d post coitum, [dpc]), and fertilized embryos were collected and cultured in potassium simplex optimization medium (KSOM), overlaid with embryo-tested mineral oil in humidified atmospheres of 5% CO_2_ at 37°C. CD-1 females were mated with vasectomized CD-1 males and used as recipients for embryo transfer at 0.5 or 2.5 dpc. Unless otherwise specified, all reagents were obtained from Sigma-Aldrich.

### Transgenic ES cell line and culture

We used the R1 ES cell line, routinely cultured on inactivated cellular feeder layers in ES cell medium, composed of Dulbecco’s modified Eagle’s medium (Gibco), supplemented with 20% fetal bovine serum (Gibco), 1% nonessential amino acid (Gibco), 0.1 mmol/L β-mercaptoethanol (Gibco), 1 mmol/L glutamine (Gibco), 1% nucleosides (Gibco), 50 units/mL penicillin, 50 μg/mL streptomycin (Gibco) and 1,000 units/mL recombinant mice leukemia inhibitory factor (Millipore). ES cells were electroporated with the linearized *pEGFP-N1* (Clontech) by using a BTX Electroporation Generator (BTX, Inc., San Diego, CA) at 250 V and 90 μs. Neomycin (Gibco) selection was performed 2 d after transfection to obtain stable transgenic ES cell lines.

### Characterization of transgenic ES cell line

#### Immunohistochemistry and AP staining

ES cells grown on feeder cells were fixed in 4% paraformaldehyde for 20 min, permeabilized with 0.2% Triton X-100 for 30 min, and blocked in 3% bovine serum albumin in phosphate-buffered saline (PBS) for 2 h. Cells were incubated with primary antibody overnight at 4°C, washed, and incubated with Alexa Fluor (Invitrogen) secondary antibody for 3 h. *Oct4* and *Sox-2* antibodies were obtained from Millipore. Alkaline phosphatase staining was done according to the manufacturer’s recommendations (Millipore).

#### Teratoma formation

ES cells were washed with PBS, trypsinized to obtain a single-cell suspension, and collected by centrifugation. 2×10^6^ cells were subcutaneously injected into immune-deficient BALB/cA-nu mice. 4 wk after injection, teratomas were dissected, rinsed once with PBS, and fixed in 10% formalin. Teratomas were embedded in paraffin, sectioned, stained with hematoxylin/eosin, and then visualized with a Olympus(IX71) microscope and photographed.

#### Expression analysis of pluripotency marker genes by RT-PCR

Reverse-transcription polymerase chain reaction (RT-PCR) was performed to evaluate the pluripotency of ES cells. Total RNA was isolated from ES cells using an RNAprep Cell/Bacteria Kit (TIANGEN) according to the manufacturer’s protocol. We subjected 0.5 μg of RNA to the RT reaction using Superscript (TaKaRa). *PrimeSTAR® HS* DNA Polymerase (TaKaRa) was used for the PCR reaction. Primers used are listed in Table 1[22].

**Table 1:** Primers used for detection of pluripotency marker genes

**Gene**	**Sequences**	**Length, bp**
*Oct4*	CTG AGG GCC AGG CAG GAG CAC GAG	485
	CTG TAG GGA GGG CTT CGG GCA CTT	
*Nanog*	AGG GTC TGC TAC TGA GAT GCT	363
	CAA CAC CTG GTT TTT CTG CCA CCG	
*Sox-2*	GGT TAC CTC TTC CTC CCA CTC CAG	193
	TCA CAT GTG CGA CAG GGG CAG	
*Rex-1*	ACG AGT GGC AGT TTC TTC TTG GGA	287
	TAT GAC TCA CTT CCA GGG GGC ACT	
*β-actin*	CCG CCA CCA GTT CGC CAT G	778
	CCG CTC GTT GCC AAT AGT GAT GAC	

#### In vitro formation of embryoid body

ES cells were washed with PBS, trypsinized, resuspended with embryoid body (EB) medium (without LIF), and 20- to 30-μL drops containing 400 to 1,000 ES cells were plated on the lid of petri dishes in regular arrays. A 100-mm dish can accommodate approximately 30 to 40 drops. The lid was inverted and placed over the bottom of a petri dish filled with PBS to prevent the drops from drying out. The petri dishes with hanging drops were incubated for 2 d. Embryoid body-like aggregates were harvested and subsequently transferred into bacterial-grade dishes and cultivated for 3 to 5 d. For further differentiation, 5 to 7-d-old EBs were plated on gelatin-coated tissue culture plates.

### Chimera production

#### Microinjection

The ES cells were injected into the mice embryos by PMM as described [17-19]. Fertilized embryos were cultured in KSOM *in vitro* until 2.0 dpc, 2.5 dpc, and 3.5 dpc for the production of 4- to 8-cell and blastocyst embryos. ES cells were trypsinized to obtain a single-cell suspension, and the cell suspension was kept on ice in 1 mL ES cell medium with HEPES in a 15-mL tube until use. Ten to fifteen ES cells were injected into the 4- and 8-cell and blastocyst embryos, injected blastocysts were cultured in KSOM at 37°C in an atmosphere of 5% CO_2_ for 1 to 2 h until the blastocyst cavity was recovered.

### Embryo transfer

Healthy microinjected 4- to 8-cell embryos were surgically transferred to the oviduct of 0.5 dpc pseudopregnant female recipients. The microinjected blastocysts were transferred to the uterine horns of 2.5 dpc pseudopregnant females using the standard procedure.

### Fluorescence microscope

*In vitro* development of 4-cell stage embryos injected with ES cells were observed with an Olympus inverted fluorescence microscope until the blastocysts hatched.

### Statistical analysis

In the efficiency of chimera production experiment, the data was processed with SPSS 17.0 (SPSS Inc., Chicago, IL) statistical software. Significance was declared at *P*≤0.05.

## Results

### Production of transgenic ES cell line

**Figure 1:**
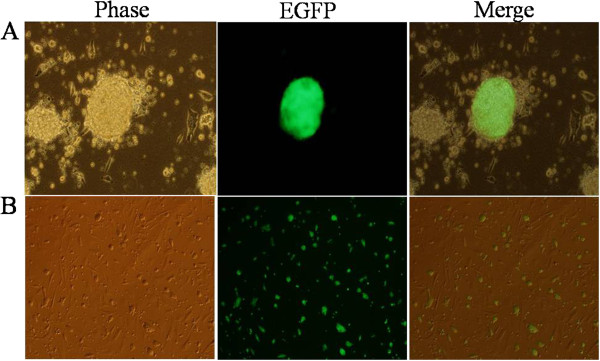
**Transgenic ES cell clones and expansion culture.** Two wk after selection, the positive and normal ES morphologic clones were picked under the fluorescence microscope (**A**). These cells were expanded and all expressed the exogenous EGFP protein (**B**).

To investigate and easily observe the *in vitro* development of chimeric embryos, we first generated the transgenic ES cell line. Linearized *pEGFP-1* was introduced into the R1 ES cell line by electroporation using the BTX (T820) electroporator. The G418 selection was performed 24 h later, and after 2 wk, selection of the positive clones was observed using the fluorescent microscope (Figure 1A). Next, the positive ES clones, which had normal morphology, were picked and expanded in culture, and successfully generated the transgenic ES cell line that expressed *EGFP* (Figure 1B).

### Characterization of transgenic ES cell line

**Figure 2:**
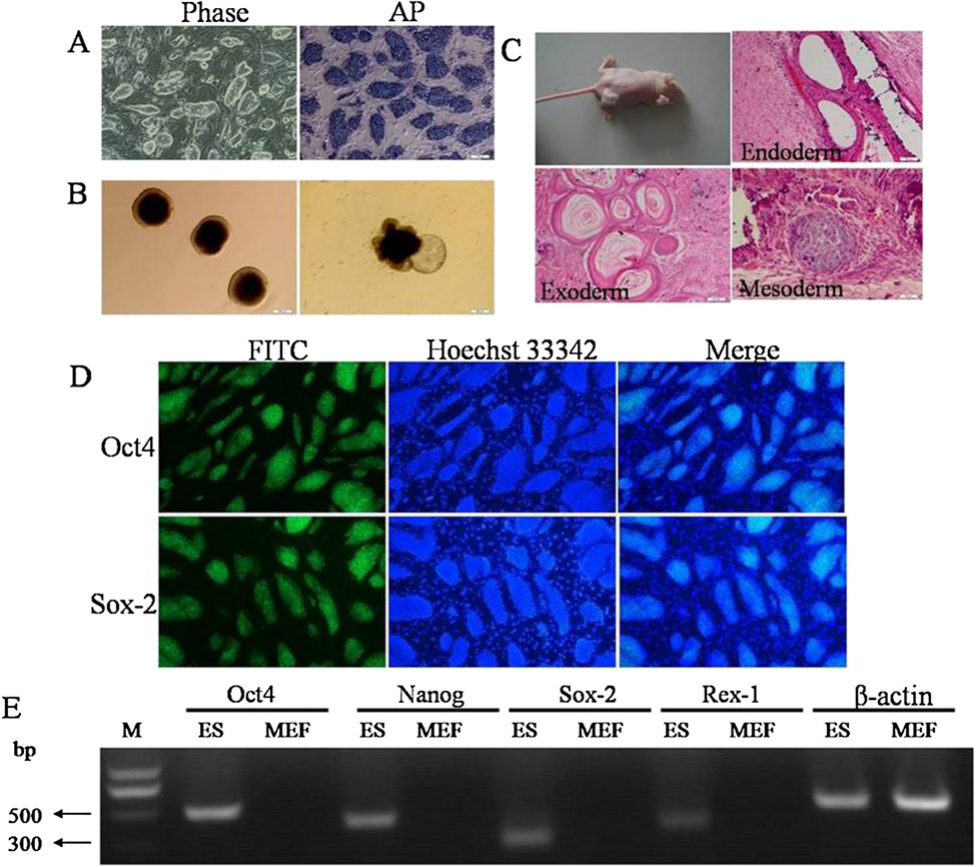
**Characterization of transgenic ES cell line.** Normal morphologic ES clones were visible under the phase-contrast microscope and stained positive for AP (**A**). Using the handing drop method, the ES cells formed the embryo-like aggregates and outgrowths after plating them onto tissue culture plates (**B**). Teratoma formation in immunodeficiency mice from injected ES cells. Hematoxylin and eosin staining was performed on the teratomas. The resulted teratomas contained tissues representing all three germ layers: endoderm, gut-like epithelium; mesoderm, cartilage; exoderm, epidermal tissues (**C**). Immunofluorescence analysis of ES cells for pluripotency markers. The clones express the embryonic markers *Oct4* and *Sox-2*. Nuclei were stained with hoechst 33342. Underlying fibroblasts provide a negative control (**D**). RT-PCR analysis of ES cell marker genes *Oct4, Nanog, Sox-2*, and *Rex-1; β-actin* was used as the loading control. M: DL 2000 DNA Marker (**E**).

To confirm that the transgenic ES cell line retained its stem cell properties, we examined them for expression of pluripotency markers. As shown in Figure 2, the ES cell clones still have normal morphology (Figure 2A, left): apparent clone boundaries, high refractivity, tight, round colonies of cells with a high nucleus to cytoplasm ratio, and stained for AP (Figure 2A, right). When the ES cells were allowed to form EBs by suspension culture, embryoid body-like aggregates were formed after five days in culture (Figure 2B, left), and we observed outgrowths after plating them onto tissue culture plates (Figure 2B, right). Next, we injected ES cells subcutaneously into immunodeficient mice. 4 wk later, upon histological examination, the ES cell line gave rise to teratomas (Figure 2C) that contained derivatives of all three germ layers, including gut-like epithelium (endoderm), cartilage (mesoderm), and epidermal tissues (exoderm). Immunocytochemistry revealed that the ES cells expressed *Oct4* and *Sox-2* (Figure 2D). RT-PCR analysis revealed that the ES cells expressed marker transcripts for *Oct4, Nanog, Sox-2* and *Rex-1* (Figure 2E). In conclusion, the transgenic ES cells retained their pluripotency.

### Generation of chimeric embryos and mice

**Figure 3:**
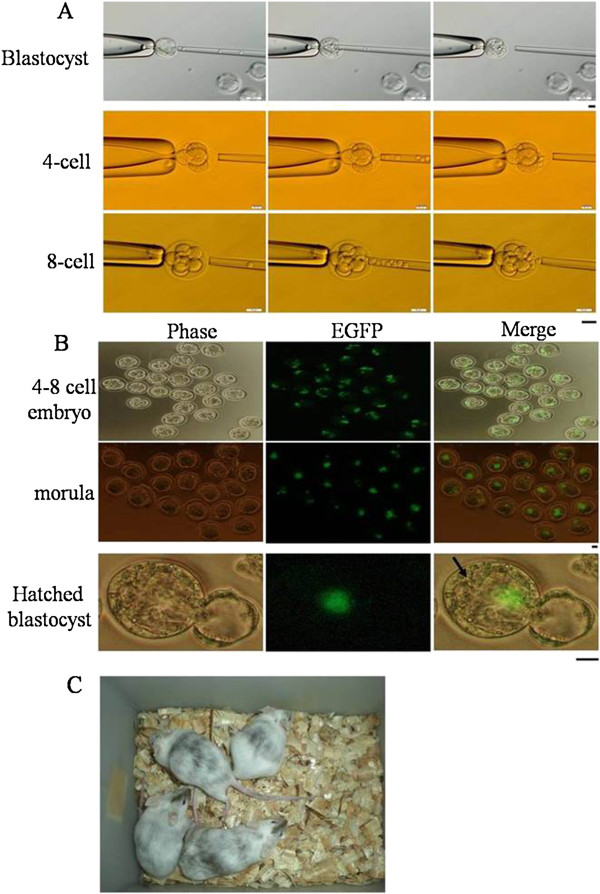
**Generation of chimeric embryos and mice using the PMM method.** Four- to eight-cell and blastocyst stage embryos were injected with 10–15 ES cells with the application of Piezo (**A**). The *in vivo* development of transgenic 4- to 8-cell stage embryos injected with ES cells was observed under the fluorescence microscope (**B**, black arrow: ICM). Chimeras were obtained from injecting the ES cells into the embryos (**C**). Scale bar = 20 μm.

To compare the production rate of chimeric mice, we injected the ES cells into the 4- to 8-cell and blastocyst stage embryos with the application of PMM (Figure 3A). The injected blastocysts were cultured in KSOM medium until the blastocele was retrieved. As shown in Table 2, the blastocyst group was higher but no significant difference was observed between it and the 4- to 8-cell group on the percentage of total born and chimeric mice. In addition, the production of male chimeric mice was higher in the latter group but no significant difference occurred. We could not yield the F0 ES cell mice based on 100% coat color. To investigate the fate of ES cells and chimeric embryo development *in vitro*, we used a fluorescent microscope to observe the injected 4- to 8-cell embryos until they hatched. We observed *EGFP* expression in the hatched blastocysts (Figure 3B), but most of the ES cells allocated to the trophectoderm layers, and had no apparent tendency to integrate into the ICM. They appeared to integrate randomly. This result was consistent with the production rate of chimeric mice.

**Table 2:** Efficiency in the production of chimeras from ES cells injected into 4- to 8-cell and blastocyst stage embryos

**Host embryo**	**No. injected embryos transferred**	**No. recipient mice**	**No. total born (%)**	**No. total chimeras (%)**	**No. total male (%)**
4- to 8- cell	248	6	27 (10.89)	12 (44.4)	9 (75)
blastocyst	618	20	80 (12.9)	42 (52.5)	28 (66.7)

## Discussion

Studies on the control of gene expression in eukaryotes based on the ability to induce the transient and stable expression in mammalian cells have contributed greatly to our current knowledge of the molecular mechanisms governing regulation of gene expression. An individual gene proved to be repaired using homologous recombination in ES cells [23] and the first genetically engineered mutant mouse was generated in 1990 [3,24] after the successful isolation of ES cell line. To date, various transfection methods have been developed to transfer particles into cells including calcium phosphate DNA precipitates [25], viral vectors [26], liposomes [27] and direct injection of genes into the nucleus. However, use of these methods is limited because: (1) they work efficiently in only limited cell types and most are adherent cells; and (2) cells transfected with these methods are subject to a high frequency of damage. Subsequently, a physical method that induces an enormous enhancement of DNA transport across cellular membranes was reported [28,29]. Briefly, using the short electric impulses above a certain field, the membrane structure was permeable and it was convenient for the material to cross without damaging it. In addition, these methods were also used in embryo fusion. We also compared electroporation and liposome-mediated transfection, and found that not only the transfection efficiency but also the quality of the positive clones was superior after electroporation versus liposomes.

Various techniques have been developed to maintain the developmental potential of ES cells *in vitro*[30] and restrict the developmental potential of host embryos to increase the efficiency of germline chimeric mice production [31,32]. Tetraploid complement was the most efficient method to generate viable and fertile ES mice completely from ES cells [33] and it's the “gold standard” to test pluripotency of the cell lines. Unfortunately, tetraploid embryos are less viable than normal embryos; mice produced by this method have nonspecific lethality and congenital abnormalities and complications in phenotypic analysis [20]. Previous observations showed that injection of ES cells at an earlier stage might increase degrees of ES cell contribution [20], and high viable germline chimeras were obtained by 8-cell embryo injection [13]. With the application of laser and PMM, F0 ES mice with full germline transmission were generated successfully [21].

In the present study, we aimed to compare the efficiency of chimeric mice production with the PMM method by injecting high passage (over 35) ES cells into the 4- to 8-cell and blastocysts embryos. Our results showed that the efficiency of chimeras did not differ between the two types of embryo injection, but all were higher than previously reported [19]. In 8-cell injection, the efficiency of chimera production and the proportion of male mice was much lower than previously reported [13]. We were unable to yield the F0 mice based on 100% coat color, which was not consistent with previous reports [13,20,21]. We traced the fate of ES cells in chimeric embryos and found that ES cells did not preferentially allocate to the ICM. This was in accordance with the data on chimera production. Reports showed that the combination of the genotypes of ES cells and host blastocysts is important for production of germline transmission chimeras, and the C57BL/6 are the suitable host for 129-derived ES cells [3,34]. In this study, ES cells were derived from 129 mice and had been cultured *in vitro* for a long time (over 35 passages). Although they retained their pluripotency, they might not be suitable for generating chimeras. Wang report that R1 cells lost their totipotency in producing viable ES mice after passage 14 and the CD-1 host embryos may have been unsatisfactory with the combination of ES cells [35].

## Conclusions

In the present study, we successfully generated a transgenic ES cell line and we gained satisfactory efficiency of chimera production by injecting into 4- to 8-cell and blastocyst embryos with the PMM method, but we found no significant difference between the two types of injection. The injected ES cells had no tendency to integrate into the ICM of the embryos based on tracing the ES cell fate. Interestingly, we yielded a higher proportion of male chimeras by injecting ES cells into 4- to 8-cell stage embryos than blastocyst injection. In addition, the PMM method is an efficient way to generate chimeric mice. These results show that these methods provide another approach to developing transgenic mice derived from ES cells.

## Competing interests

The authors declare that they have no competing interests.

## Authors’ contributions

MH carried out the experiments and drafted the manuscript. SZ advised MH in performing the study and writing the manuscript for publication. WH, JZ, YB, FG and LL provided a lot of help with these experiments. All authors read and approved the final manuscript.

This study was supported by the National Basic Research and Development Program of China (973 Program; No. 2011CB944202, 2010CB945001, and 2009CB941601) and the National Science Supporting Plan of China (2011BAD19B03).
